# Strategies to Reduce Hospital Length of Stay: Evidence and Challenges

**DOI:** 10.3390/medicina61050922

**Published:** 2025-05-20

**Authors:** Rahim Hirani, Dhruba Podder, Olivia Stala, Ryan Mohebpour, Raj K. Tiwari, Mill Etienne

**Affiliations:** 1 School of Medicine, New York Medical College, Valhalla, NY 10595, USA; 2 Graduate School of Biomedical Sciences, New York Medical College, Valhalla, NY 10595, USA; 3 Department of Neurology, New York Medical College, Valhalla, NY 10595, USA

**Keywords:** hospital length of stay, nosocomial infections, predictive modeling, patient outcomes, healthcare efficiency

## Abstract

Hospital length of stay (HLOS) is a critical healthcare metric influencing patient outcomes, resource utilization, and healthcare costs. While reducing HLOS can improve hospital efficiency and patient throughput, it also poses risks such as premature discharge, increased readmission rates, and potential compromise of patient safety. This literature review synthesizes current evidence on the determinants of HLOS, including patient-specific factors such as demographics, comorbidities, and socioeconomic status, as well as hospital-related factors like admission route, resource allocation, and institutional policies. We also examine the relationship between HLOS and key clinical outcomes, including mortality, readmission rates, and healthcare-associated infections. Additionally, we evaluate predictive modeling approaches, including artificial intelligence and machine learning, for forecasting HLOS and guiding early intervention strategies. While interventions such as enhanced recovery after surgery (ERAS) protocols, multidisciplinary care teams, and structured discharge planning have demonstrated efficacy in reducing HLOS, their success varies based on healthcare setting, patient complexity, and resource availability. Predictive analytics, incorporating clinical and non-clinical variables, offer promising avenues for improving hospital efficiency, yet may carry risks related to data quality and model bias. Given the impact of HLOS on clinical and economic outcomes, targeted interventions and predictive models should be applied cautiously, with future research focusing on refining personalized discharge strategies and addressing disparities across diverse patient populations.

## 1. Introduction

### 1.1. Importance of HLOS as a Metric

Hospital length of stay (HLOS) has long been considered a critical metric in evaluating healthcare quality, operational efficiency, and patient outcomes [1]. As healthcare systems strive to optimize resource allocation and enhance patient care, understanding the factors influencing HLOS and its implications is essential. The significance of HLOS extends beyond merely quantifying time spent in the hospital; it serves as a surrogate indicator of the severity of illness, hospital resource utilization, and the quality of discharge planning and post-discharge care [2]. Notably, HLOS is intricately linked to various health outcomes, such as morbidity, mortality, readmissions, and long-term recovery. Recent studies suggest that patients from lower socioeconomic backgrounds and minority populations are disproportionately affected by prolonged HLOS, encountering barriers to timely discharge and adequate outpatient support [3,4]. Addressing these disparities is critical for optimizing HLOS and promoting equitable care delivery.

One might assume that longer hospital stays allow for more comprehensive care and better patient recovery. While this may be true for patients with complex comorbidities or severe conditions requiring extended care, prolonged HLOS is often associated with increased risks, including nosocomial infections and healthcare costs [5,6]. Therefore, it is crucial to balance the need for adequate care with the risks associated with extended hospitalizations. For instance, research has shown that in specific conditions, early discharge, when accompanied by proper follow-up and outpatient support, can enhance recovery while minimizing hospital-related complications [5,6].

HLOS is frequently used as a benchmark for hospital performance and a proxy for the quality of clinical care. Prolonged stays can indicate delays in treatment, complications, or inefficient hospital operations, while shorter stays may signal premature discharges or suboptimal patient management [2,7]. The complexity of interpreting HLOS underscores the need for context-sensitive analysis. For example, an unexpectedly short HLOS in patients with complex conditions may indicate gaps in comprehensive care or inadequate follow-up.

Along with predicting hospital and ICU mortality, HLOS also serves as a surrogate marker for healthcare resource utilization, impacting overall costs for patients. There has been growing interest in analyzing HLOS as a variable that correlates with various outcomes to improve hospital performance [8,9].

Moreover, HLOS is associated with patient satisfaction [8] and healthcare costs [9]; longer stays often lead to higher healthcare expenditures and increased financial strain for both institutions and patients. In value-based care frameworks, hospitals are incentivized to reduce unnecessary HLOS to improve efficiency without compromising patient safety [10]. However, efforts to reduce HLOS must be balanced with the risk of early discharges, which can lead to avoidable readmissions and adverse outcomes [11].

Beyond individual patient outcomes, HLOS serves as a population health metric that reveals broader disparities in healthcare access and delivery. Studies indicate that patients from socioeconomically disadvantaged backgrounds and minority populations experience prolonged HLOS due to barriers in discharge planning, inadequate outpatient support, and limited access to follow-up care [3,12]. Addressing these disparities is essential for optimizing resource allocation and promoting equitable care delivery.

### 1.2. Ethical Considerations and Predictive Models

The use of predictive models, including artificial intelligence (AI) and machine learning, has gained traction in estimating HLOS and informing discharge planning. While AI-driven tools offer potential benefits in forecasting patient outcomes and optimizing resource use, they also pose ethical challenges. Recent studies highlight concerns related to data quality, model bias, and the risk of applying predictive analytics without adequate contextualization [13,14]. For instance, AI models trained on biased data may disproportionately impact vulnerable populations, leading to disparities in care. Therefore, integrating demographic variability and social determinants of health into predictive models is essential for equitable healthcare [13,14].

Hospitals participating in bundled payment methods, such as Medicare’s BPCI-A (Bundled Payments for Care Improvement-Advanced), are motivated to reduce HLOS while maintaining quality. However, hospitals must carefully manage shorter stays to avoid increased post-discharge costs, such as readmissions or prolonged post-acute care [13,14]. Enhanced recovery protocols, multidisciplinary collaboration, and structured discharge planning are among the strategies to manage HLOS without compromising care quality [14].

### 1.3. Historical and Global Perspectives

Historically, HLOS has decreased significantly over the past several decades due to advancements in medical technology, early mobilization strategies, and enhanced recovery programs [15,16,17]. In the 1960s, the average HLOS in U.S. hospitals was approximately 21 days, which decreased to seven days by the early 2000s [18]. This reduction reflects improved surgical techniques, more effective pharmacological interventions, and a shift toward outpatient procedures. The introduction of minimally invasive surgical techniques, such as laparoscopic surgeries, has also significantly shortened recovery times and HLOS [19].

Internationally, significant variations exist in the average HLOS, reflecting differences in healthcare systems, clinical practices, and socioeconomic factors. Countries with robust primary care systems and integrated care pathways tend to report shorter hospital stays compared to those with fragmented systems [20]. For example, the average HLOS in the United States is often higher for certain conditions compared to European nations, indicating disparities in care coordination and discharge protocols.

Siddique et al. (2021) found that hospital-led interventions such as discharge planning, geriatric assessment, and interdisciplinary care teams can reduce HLOS in high-risk populations, although the effectiveness varies across different patient groups [21]. Fernandez et al. (2018) demonstrated that better coordination between hospitals and social care services in England can significantly reduce post-operative HLOS, emphasizing the importance of integrated care pathways [22]. Kutz et al. (2022) noted that shorter HLOS is not necessarily associated with adverse outcomes if discharge processes are well-managed, further supporting the role of effective discharge planning in reducing hospital stays [23]. In the United Kingdom, nationalized health services have implemented streamlined discharge plans that contribute to shorter HLOS for conditions such as elective surgery and chronic disease management, suggesting that policy-driven interventions can play a significant role. These findings collectively underscore the importance of integrated care pathways and effective discharge planning in reducing HLOS and improving patient outcomes.

In low- and middle-income countries, challenges such as limited healthcare infrastructure, inadequate staffing, and delayed access to specialized diagnostics contribute to prolonged HLOS [20]. Structural barriers such as resource limitations and staffing shortages further exacerbate these challenges, underscoring the need for targeted interventions. Antimicrobial stewardship programs, for instance, have been associated with a 19.1% reduction in HLOS in LMIC hospitals, demonstrating the potential of structured interventions to optimize hospital resources [24]. Additionally, programs aimed at improving hospital discharge practices through home visits and multidisciplinary team models have been shown to effectively reduce readmission rates and associated HLOS in low-income settings [25]. Addressing these disparities requires healthcare reforms that not only improve resource allocation but also adapt successful LMIC-specific strategies to broader contexts.

### 1.4. Objective of the Review

While substantial research has identified factors associated with prolonged HLOS, such as comorbidities, hospital resources, and discharge planning, less is known about how predictive models can be effectively integrated into patient-centered care to ensure equitable outcomes. Additionally, the application of real-time data to dynamically adjust HLOS predictions remains underexplored, particularly in the context of vulnerable populations with complex care needs

This review aims to provide a comprehensive analysis of the relationship between HLOS and patient outcomes, with a specific emphasis on predictive modeling, interventions, and economic impacts. We will explore evidence from recent studies to elucidate the impact of prolonged HLOS on nosocomial infections, readmissions, and healthcare costs, while also identifying key predictors of extended stays. Additionally, we will discuss innovative interventions, such as ERAS protocols and multidisciplinary care models, that have demonstrated efficacy in reducing HLOS without compromising patient safety. By synthesizing current evidence, this review will also highlight the economic implications of prolonged stays and assess the cost-effectiveness of targeted interventions.

In addition to providing a comprehensive analysis of the factors influencing HLOS, this review will utilize a systems theory framework to organize the diverse determinants of HLOS and their interrelationships. This framework will facilitate a more structured synthesis of evidence, highlighting how predictive modeling, clinical interventions, and economic considerations intersect to impact HLOS. By adopting a systems-oriented perspective, we aim to identify key pathways that contribute to prolonged stays, underscore potential intervention points, and suggest areas for future research within a value-based care context.

In summary, optimizing HLOS is crucial for improving patient outcomes and healthcare system performance. By clearly defining the scope and focus areas—predictive analytics, clinical interventions, and economic impacts—this review aims to inform clinical practice and guide future research in this critical area of health services research.

While this review aims to provide a comprehensive analysis of HLOS and its associated clinical and economic outcomes, it is important to acknowledge the limitations inherent in a narrative synthesis. Due to the heterogeneity of study designs, sample sizes, and patient populations included in the selected literature, the findings should be interpreted with caution. The absence of a quantitative meta-analysis or systematic review protocol may limit the generalizability of the conclusions. Nevertheless, this review synthesizes key trends and emerging patterns to offer a broader perspective on HLOS determinants and potential intervention strategies, laying the groundwork for more targeted and systematic analyses in future research.

### 1.5. Methods

This narrative review was conducted to synthesize current evidence on HLOS, its determinants, and associated clinical and economic outcomes. A comprehensive literature search was carried out in PubMed, Scopus, Web of Science, and Cochrane Library using search terms including “hospital length of stay”, “predictive modeling”, “discharge planning”, “readmissions”, “enhanced recovery”, “nosocomial infections”, and “hospital resource utilization”. The search was limited to articles published in English between January 2013 and March 2025 to ensure the inclusion of recent and relevant studies.

The inclusion criteria encompassed studies that examined HLOS as a primary or secondary outcome, with a particular focus on patient-specific factors such as age, comorbidities, and socioeconomic status, as well as hospital-related factors like discharge planning, predictive models, and economic impacts of prolonged stays. Eligible study designs included systematic reviews, meta-analyses, cohort studies, randomized controlled trials, and large observational studies with a sample size of at least 100 participants.

Studies were excluded if they were case reports, opinion pieces, or small sample studies (*n* < 100). Additionally, studies focusing exclusively on pediatric populations or highly specific disease cohorts without broader implications for HLOS were omitted. Studies lacking clear HLOS data or failing to distinguish between in-hospital and post-discharge outcomes were also excluded to maintain consistency in data extraction and synthesis. The initial screening and data extraction were conducted by the lead author. To minimize bias, inclusion and exclusion criteria were defined a priori in consultation with co-authors, and key decisions regarding study eligibility were revisited during manuscript drafting.

Data extraction focused on HLOS duration, primary predictors, intervention strategies, and associated clinical outcomes, including nosocomial infections, readmissions, and healthcare costs. The data were synthesized thematically to align with the review’s objective of identifying effective strategies to optimize HLOS while maintaining patient safety and minimizing the economic burden. Studies were further categorized by intervention type, such as ERAS protocols and predictive modeling, as well as by population characteristics, including elderly patients and socioeconomically disadvantaged groups. While a PRISMA flow diagram was not used due to the narrative nature of this review, methodological transparency was prioritized through clear inclusion criteria, structured thematic synthesis, and reflexive screening procedures

## 2. Factors Influencing HLOS

Before we dive into how and why HLOS affects patient outcomes, it is important to understand some of the factors that confer extended HLOS for some patients. Patient-specific factors such as patients’ age, history of previous admission, marital status, type of treatment, and employment play a major role [26]. Other patient-related factors have also been shown to significantly influence HLOS. For instance, elderly patients with multiple chronic conditions often require longer stays due to the complexity of their care needs [26]. Studies have highlighted that preoperative conditions, such as uncontrolled diabetes or poor functional status, can prolong recovery times and increase the likelihood of post-surgical complications [27]. Patients with limited financial resources or inadequate support systems may experience delayed discharges due to the lack of appropriate follow-up care or safe living conditions [11]. Research has shown that patients with strong family support systems and access to outpatient rehabilitation services have shorter recovery periods and fewer post-discharge complications compared to those without these resources [28]. It has also been observed that increased patient complications, higher injury severity, and negative experience with physicians or staff can also affect HLOS for a patient [21,29].

Conversely, a myriad of factors unrelated to patients’ conditions or self-made decisions have been identified as impacting HLOS. External factors, including hospital bed availability, staff-to-patient ratios, and the efficiency of diagnostic services, also play a crucial role. Inefficiencies such as delays in diagnostic testing or discharge planning can prolong HLOS and negatively impact patient outcomes [30]. Additionally, staffing shortages, particularly in critical care and specialty units, can delay necessary interventions and prolong recovery times [9]. Factors such as availability of beds, resources to manage that particular ailment, social care or community nursing support, and hospital management style also contribute to prolonged HLOS [8,30]. Addressing these systemic issues requires coordinated efforts to optimize resource distribution, implement predictive analytics to anticipate discharge needs, and standardize care pathways to reduce variability in HLOS.

Many studies have shown how age can impact the average HLOS, which is codependent on the nature of the injury/trauma. The extent of the injury informs whether additional surgeries are required and thus can also increase the overall HLOS. For example, a study in which patients who were emergently admitted with gastroparesis were studied retrospectively showed that adults spent an average of 4.46 days in the hospital when they did not have surgery and 11.33 days when they had surgery. In comparison, the elderly spent an average of approximately 6 days when they did not have surgery and 13 days when they had surgery [31]. This lengthy stay, in turn, can increase the average cost of care. In the same study, elderly patients spent approximately USD 5000 and USD 3000 more as compared to their adult counterparts in the absence and presence of an operation, respectively [31]. Additionally, longer HLOS has been associated with an increased risk of mortality in certain injuries. For example, Elgar et al. showed that for every additional day patients with blunt thoracic trauma stayed in the hospital, the odds of mortality increased by 9% [32].

Furthermore, longer HLOS is associated with an increased risk of mortality in various other ailments, such as patients emergently admitted with hemorrhoids, chronic duodenal ulcers, ventral hernias, appendicitis, or oropharyngeal dysphagia [33,34,35,36,37]. This increased mortality associated with an increase in HLOS may be attributed to several factors; however, nosocomial infections are among the most significant.

In a prospective cohort study, Murni et al. concluded that HLOS > seven days can significantly increase the odds of nosocomial infections [38]. This is specifically concerning given the fact that in many emergency cases, patients spend more than seven days in the hospital awaiting a full recovery. According to the Centers for Disease Control (CDC), in the United States, there are approximately 1.7 million cases of nosocomial infections, and 99,000 deaths associated with these infections, each year, making it a national healthcare priority [39].

Nutrition can also play an important role in affecting overall HLOS. In a prospective, analytical cohort study of 161 trauma patients, malnutrition was found to be an independent risk factor for morbidity, which consequently increased HLOS and mortality [40]. Nigatu et al., in their prospective cohort study, found HLOS to be two times longer in malnourished patients as compared to their well-nourished counterparts (17.2 days vs. 8.3 days) [41]. Yet, another study concluded that malnourished patients are approximately 75% more likely to have in-hospital mortality and 105% more likely to have longer HLOS [42]. These results pose some important questions and implications regarding how patients are monitored during the intake and throughout their stay. Perhaps more studies and measures are necessary to identify if the patients are malnourished earlier in their hospital admission.

Several key studies have examined the factors influencing HLOS across diverse patient populations and healthcare settings. These studies highlight the impact of demographic, clinical, and systemic variables on hospitalization duration. For instance, age, gender, and comorbidities consistently emerge as significant determinants, with older adults and patients with multiple chronic conditions often experiencing prolonged stays. Similarly, hospital-specific factors such as admission route, operative management, and institutional policies also contribute to variations in HLOS. The following table summarizes key findings from recent research on HLOS determinants, outlining the primary factors studied, their impact on hospital stay duration, and statistical significance (Table 1).

The studies summarized in Table 1 illustrate consistent trends in HLOS determinants. Advanced age and the presence of comorbidities are strongly correlated with longer hospital stays across various medical conditions, emphasizing the need for early interventions to mitigate complications. Socioeconomic and hospital-related factors, such as insurance type, admission status, and institutional resources, also play a critical role in determining hospitalization duration. Additionally, targeted interventions—such as early analgesia in elderly trauma patients and outpatient preoperative evaluations for surgical patients—have been shown to reduce HLOS significantly. These findings underscore the importance of personalized care strategies, proactive discharge planning, and efficient hospital resource allocation in optimizing patient outcomes and minimizing unnecessary hospital stays.

### 2.1. Impacts of Extended HLOS

HLOS is a critical factor influencing patient outcomes, healthcare efficiency, and hospital resource allocation. While prolonged hospitalization is sometimes necessary for managing complex conditions, extended stays are associated with an increased risk of nosocomial infections, higher mortality rates, worsened post-discharge quality of life, increased hospital readmissions, and significant financial burdens. Understanding these associations is crucial for optimizing patient care and reducing unnecessary hospitalization.

Why is a longer HLOS associated with worse patient outcomes, despite the extensive resources available in hospitals? One intuitive explanation is that the sooner a patient receives the necessary medical care, the quicker their recovery and the lower their risk of complications are. This principle has been demonstrated across various conditions. For instance, in orthopedics, fast-track protocols following total knee replacement have been shown to accelerate post-surgical recovery [55]. Early rehabilitation and mobilization efforts increase the likelihood of greater walking distance at discharge [28], while prompt initiation of clinical care is linked to faster recovery after a concussion [56].

One of the most concerning consequences of prolonged hospital stays is the increased risk of nosocomial, or hospital-acquired, infections, which contribute to greater morbidity, mortality, and financial costs. Patients with prolonged HLOS have a significantly higher likelihood of developing infections such as urinary tract infections (UTIs), bloodstream infections (BSIs), surgical site infections (SSIs), and pneumonia. Studies indicate that hospital-acquired infections (HAIs) prolong hospitalization by an additional 3.9 to 6.6 days, depending on the type of infection, with pneumonia contributing to the most substantial delay (6.6 days) (Table 2) [57].

The presence of nosocomial infections is also strongly correlated with increased mortality. Pittet et al. (1994) found that patients who developed nosocomial bloodstream infections had an extended hospital stay of 24 days, with a 50% crude mortality rate and an attributable mortality rate of 35% (95% CI, 25–45%) [58]. Similarly, Chen et al. (2005) [59] reported that patients with nosocomial infections had a median hospital stay 18.2 days longer than uninfected patients (*p* < 0.001), highlighting the significant burden of infections on patient outcomes. Efforts to reduce HLOS by improving early discharge planning and infection control strategies could mitigate these risks [59].

Such nosocomial infections can, unfortunately, confer longer HLOS and create a vicious positive feedback loop. Jia et al., in their case-control study of 2119 patients across 68 hospitals in China, reported that hospital-associated infections caused an increase in HLOS of approximately 11 days [60]. Furthermore, pneumonia and urinary tract infection are the two most common complications in several ailments, which can extend the HLOS anywhere from 161% to 281%, depending on their comorbidities and demographics [61]. This not only puts a strain on patients’ health but also brings additional financial burden, especially if they are underinsured or uninsured [10,62]. Lastly, patient experience in the hospital with their medical team and staff can dictate an individual’s state of mind, which in turn can impact recovery. This patient experience was also shown to be translated to their behavior, as it pertains to their health, for example, regarding adherence to recommended clinical practice and medication [11]. While there are several factors for which we know the mechanistic details, others are not so clear. For example, a retrospective cohort study of 8291 patients who had undergone appendectomy or laparoscopic cholecystectomy showed that a higher preoperative blood glucose level was significantly associated with an increased HLOS [27]. While the association between elevated preoperative blood glucose and prolonged HLOS is evident, this may be partially explained by known links between hyperglycemia and worse outcomes, including increased in-hospital mortality and complications in both diabetic and non-diabetic patients [63]. However, the specific mechanisms underlying this association in the context of surgical recovery remain incompletely understood and warrant further investigation across different procedures.

Beyond infections, HLOS itself is independently linked to higher mortality rates. Kaboli et al. (2012) observed that reducing HLOS from 5.44 to 3.98 days was associated with a decline in 30-day mortality from 6.4% to 4.8% and 90-day mortality from 11.46% to 9.35% (*p* < 0.001) [17]. This suggests that prolonged hospitalization may expose patients to further complications, such as deconditioning, thromboembolic events, and cognitive decline, increasing their risk of mortality.

It is also worth noting that a longer HLOS can also have a negative impact on hospitals. For example, increased HLOS for patients leads to fewer hospital discharges, which translates to decreased profit and revenues, along with less efficient bed management [64,65]. Furthermore, it can also increase communication errors among staff and patients, leading to hospital harm [66]. This possibility of hospital harm, in turn, increases the HLOS even further. Tessier et al., in their population-based retrospective cohort study using health administrative databases, concluded that hospital harm put an additional toll of CAD 1,088,330,376 on their healthcare system in 2017 [9].

### 2.2. Quality of Life and Hospital Readmissions

While direct measures of quality of life following prolonged HLOS were not well-reported in the studies reviewed, hospital readmission rates serve as a valuable surrogate marker for post-discharge patient outcomes. Longer hospital stays are often associated with functional decline, particularly in older adults, where extended immobility leads to muscle wasting, physical deconditioning, decreased mobility, and increased dependence on rehabilitation services post-discharge.

Readmission data suggest that patients who required hospital readmission within 30 days had an initial HLOS of 6.87 days, compared to 5.18 days for those who were not readmitted (*p* < 0.001) [67]. However, Kaboli et al. (2012) found that reducing HLOS from 5.44 to 3.98 days did not lead to increased readmission rates, suggesting that prolonged hospitalization does not necessarily prevent post-discharge complications [17]. These findings indicate that excessive hospital stays may not always be beneficial, and that early discharge strategies, coupled with proper outpatient follow-up, can help maintain positive patient outcomes.

Beyond readmission risk, hospital-acquired delirium is a significant issue in patients with prolonged stays, particularly among the elderly. Patients with extended hospitalization often experience cognitive decline due to disorientation in the hospital setting, frequent nighttime disturbances, and medication side effects. Given the absence of quantitative quality of life metrics in the reviewed studies, future research should prioritize long-term functional and psychosocial outcomes of patients experiencing extended hospital stays.

### 2.3. Economic Burden of Prolonged Hospitalization

The financial impact of prolonged hospitalization is considerable, affecting both hospitals and patients. Nosocomial infections and extended HLOS lead to substantial increases in hospital costs, often burdening uninsured and underinsured populations.

Chen et al. (2005) found that nosocomial infections significantly increase hospitalization costs, adding a median of USD 6369 per hospital stay, with total expenses rising from USD 10,354 to USD 16,723 per patient [59]. Further cost analysis by Glied et al. (2016) highlighted the infection-specific financial burden, demonstrating that urinary tract infections (UTIs) contribute an additional USD 41,715 per patient, while bloodstream infections (BSIs) increase costs by USD 34,394 [57]. Surgical site infections (SSIs) result in an even greater financial impact, adding USD 69,626 per case, and pneumonia incurs the highest additional cost, at USD 78,585 per patient. These findings underscore the substantial economic burden of healthcare-associated infections (HAIs) and emphasize the need for infection prevention strategies to mitigate excessive hospital costs.

These findings underscore the need for cost-effective infection prevention measures, particularly in hospitals serving uninsured patients. Patients with bloodstream infections incur an additional USD 40,000 in costs per survivor, further demonstrating the financial burden associated with HAIs [58]. Additionally, Ward et al. (2021) found that HLOS outliers—patients staying significantly longer than expected—incurred an additional USD 77,228 per stay [68]. To further illustrate the economic burden of prolonged HLOS across diverse patient populations and healthcare settings, Table 3 summarizes findings from key studies quantifying the financial impact of specific conditions, such as nosocomial infections and HLOS outliers, on hospitalization costs. Beyond direct hospital costs, prolonged hospital stays also lead to lost wages and increased reliance on post-acute care services, such as skilled nursing facilities and home health care, contributing to the overall economic impact. Reducing unnecessary hospitalizations and implementing cost-effective discharge planning could significantly decrease these expenses while improving patient outcomes. Table 4 summarizes some recent studies depicting the impact of various medical conditions on HLOS and associated cost increases, with findings indicating significant variations across different patient populations and healthcare settings.

It can be concluded that extended HLOS is closely linked to adverse patient outcomes, including increased risk of nosocomial infections, higher mortality rates, worsened post-discharge recovery, and greater financial burdens on both patients and healthcare systems. The cyclical nature of prolonged hospitalization—where extended stays lead to complications that further lengthen hospitalization—highlights the need for strategies that promote early discharge without compromising patient care, with the utilization of predictive models emerging as a promising tool to do so.

### 2.4. Predictive Models for HLOS

A variety of predictive models have been employed to estimate HLOS, particularly at the time of admission, to prevent complications and enable early interventions. Among them, machine learning and deep learning have gained prominence for their ability to process large datasets and provide actionable predictions [20,82,83]. These approaches leverage historical patient data to predict patient outcomes, recommended treatments, and future wellness, influencing treatment strategies and decisions regarding HLOS [84].

Several predictive models have been compared in recent literature, each demonstrating varying degrees of accuracy based on patient population and clinical context. For instance, the Random Forest model has been shown to outperform other machine learning models, particularly in cardiac patients, achieving high predictive accuracy [83]. Similarly, the Artificial Neural Network model, when trained on pre-incision factors, has been noted for its superior accuracy in predicting HLOS following cardiac surgery [85]. Additionally, the Cubist method has demonstrated more effective predictions using variables available at the time of admission, suggesting it may be particularly useful for initial triage and early intervention [82].

Despite these comparative findings, existing studies indicate that no single model consistently provides optimal predictive accuracy across all patient populations and clinical conditions. Variability in patient complications, data inputs, and study design contribute to these differences, underscoring the need for further research to identify model-specific strengths and limitations. Future investigations should focus on direct head-to-head comparisons between models using standardized datasets to better inform clinical decision-making and policy development. Integrating predictive analytics into policy frameworks offers an opportunity to align financial incentives with clinical outcomes. For example, value-based reimbursement models could leverage predictive models to identify high-risk patients at admission and allocate resources for targeted interventions that prevent prolonged stays. Policymakers could also consider developing guidelines that incorporate predictive analytics into discharge planning, particularly for socioeconomically disadvantaged populations where prolonged HLOS is more prevalent. By establishing policy levers that incentivize early intervention based on predictive analytics, healthcare systems can reduce unnecessary hospital stays while optimizing resource allocation.

## 3. Factors for Reducing HLOS

**Figure 1:**
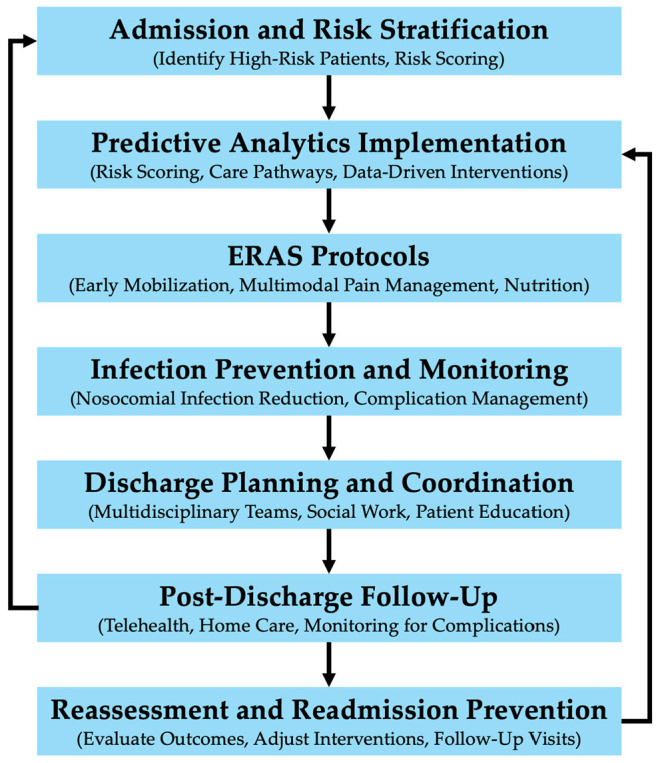
Comprehensive intervention workflow for reducing hospital length of stay (HLOS) and mitigating hospitalization costs.

Reducing HLOS without compromising patient outcomes has become a primary focus in modern healthcare. A wide range of interventions has been studied and implemented, from enhanced recovery programs to more efficient discharge processes. These interventions not only help hospitals optimize resource use but also minimize risks such as nosocomial infections and preventable readmissions. Policy initiatives could further support these interventions by implementing reimbursement reforms that reward hospitals for reducing preventable complications and HLOS through evidence-based protocols such as ERAS. Additionally, establishing standardized metrics for discharge planning and post-discharge follow-up could incentivize hospitals to adopt predictive analytics, ensuring that high-risk patients receive timely, cost-effective care while minimizing unnecessary HLOS. Figure 1 illustrates a comprehensive intervention workflow for managing HLOS, integrating predictive analytics, infection prevention, ERAS protocols, and post-discharge planning to effectively reduce hospitalization duration and associated costs. This section explores the most promising interventions, including enhanced recovery after surgery (ERAS) protocols, multidisciplinary teams, outpatient versus inpatient decision-making, and discharge planning with post-discharge follow-up.

The intervention workflow outlined in this figure presents a systematic approach for optimizing HLOS through targeted interventions at each stage of the patient care continuum. Starting with admission and risk stratification, the workflow leverages predictive analytics to identify high-risk patients and implement tailored ERAS protocols and infection prevention measures. Discharge planning is facilitated by multidisciplinary teams, incorporating social work, patient education, and resource allocation to reduce avoidable readmissions. Post-discharge follow-up through telehealth and home health services is essential for monitoring complications and ensuring continuity of care. Feedback loops indicate points where complications may necessitate re-evaluation and potential readmission, underscoring the importance of ongoing reassessment and data-driven intervention adjustments.

### 3.1. Enhanced Recovery After Surgery (ERAS) Protocols

Enhanced Recovery After Surgery (ERAS) pathways represent a comprehensive, evidence-based approach to optimizing perioperative care and accelerating recovery. These protocols have been particularly effective in procedures such as colorectal and orthopedic surgeries, where faster recovery is associated with significant reductions in HLOS and healthcare costs.

A core component of ERAS protocols is early mobilization, which encourages patients to ambulate within hours of surgery to prevent complications like deep vein thrombosis and pneumonia. Studies show that patients mobilized early after colorectal surgery experience shorter hospital stays by 1.5–3 days on average compared to those following traditional protocols [86]. Similar reductions have been observed in orthopedic procedures. One study observed that early mobilization after surgery was associated with a 1.9-day reduction in HLOS for patients undergoing total hip and knee replacements [87]. Early ambulation was associated with faster functional recovery, reduced dependency on pain medications, and increased patient satisfaction. The combined findings underscore that mobilizing patients early not only prevents complications but also improves long-term outcomes by promoting muscle strength and reducing hospital-related deconditioning. As a result, early mobilization remains an essential element of ERAS protocols across surgical specialties.

Another critical element of ERAS protocols is multimodal pain control, which aims to optimize pain management while reducing the use of opioids and their associated side effects, such as respiratory depression, nausea, and constipation. This approach typically combines regional anesthesia (e.g., peripheral nerve blocks) with non-opioid analgesics, including nonsteroidal anti-inflammatory drugs (NSAIDs), acetaminophen, and local anesthetics. Studies have shown that patients who receive multimodal pain management experience better postoperative pain relief, faster mobilization, and reduced HLOS compared to those managed with opioids alone. A 2019 study found that multimodal analgesia is associated with shorter hospital stays, with patients receiving such regimens experiencing a 0.36-day reduction in HLOS compared to those receiving opioids alone [88]. Additionally, a 2022 study examining a standardized postoperative multimodal analgesia pathway in cardiothoracic surgery patients reported reduced opioid consumption and related complications, contributing to enhanced recovery [89]. These findings underscore the efficacy of multimodal pain management in improving patient outcomes and support its integration into ERAS protocols.

The success of ERAS programs is attributed to their holistic approach, which addresses various recovery factors simultaneously, including nutrition, pain management, and physical activity. However, some heterogeneity in outcomes remains, particularly among high-risk or elderly populations, suggesting that ERAS protocols may require tailoring to individual patient needs.

### 3.2. Multidisciplinary Teams

The implementation of multidisciplinary teams has been shown to be another effective intervention for reducing HLOS. These teams typically include physicians, nurses, pharmacists, physical therapists, and other specialists who collaborate to provide comprehensive care tailored to each patient. By coordinating care, these teams can minimize delays in diagnostics, treatment, and discharge processes.

Multidisciplinary rounds (MDRs) have shown considerable success in optimizing care transitions and shortening HLOS. For instance, one study found that implementing structured MDRs reduced HLOS by 0.8 days on average while also improving patient satisfaction and clinical outcomes [90]. Another study observed that in heart failure patients, multidisciplinary rounds were associated with a significant reduction in 30-day readmission rates, suggesting that these rounds improve care continuity beyond the hospital setting [91].

Additionally, the Multidisciplinary Staffing Model, especially in critical care units, ensures that necessary interventions are promptly administered, reducing the risk of prolonged hospitalization. Suarez et al. demonstrated that integrating a specialized neurocritical care team, including a full-time neurointensivist, led to a reduction in both hospital mortality and HLOS [92]. This further supports the established relationship between HLOS and mortality, reinforcing the need for well-structured, interdisciplinary collaboration in critical care settings.

However, systematic reviews have yielded mixed findings regarding the effectiveness of multidisciplinary approaches across patient populations. A review by Mercedes et al. (2016) reported inconclusive evidence for the impact of MDRs in acute care settings, indicating that their success may depend on the complexity of the patient’s condition and the specific design of the intervention [93]. Conditions like post-surgical recovery and critical care may benefit more from MDRs than general medical conditions. Further study is needed to determine which patient populations derive the greatest benefit and how MDRs can be optimized for different clinical settings [93,94].

### 3.3. Outpatient vs. Inpatient Decision-Making

Accurate decision-making regarding whether a given patient should be treated in an inpatient or outpatient setting is crucial for optimizing HLOS. It has been demonstrated that outpatient preoperative evaluations and same-day admissions can significantly reduce hospital stays without adversely affecting patient outcomes.

One example comes from vascular surgery, where outpatient preoperative assessments have been shown to reduce postoperative HLOS by up to 5 days for certain procedures [95]. They observed that same-day admission following outpatient preoperative evaluations reduced the average HLOS from 7 to 2 days in patients undergoing carotid endarterectomy and lower extremity revascularization. The ability to complete diagnostic tests, patient education, and risk stratification before hospital admission ensures that patients receive streamlined care upon arrival and minimizes unnecessary delays.

However, not all patients are suitable for outpatient care, particularly those with multiple comorbidities or complex surgical needs. Establishing clear criteria for determining inpatient versus outpatient status is essential. For example, tailoring protocols based on preoperative risk assessments can help identify patients who may be safely managed as outpatients while flagging those who require close monitoring.

### 3.4. Discharge Planning and Post-Discharge Care

Effective discharge planning is a cornerstone of HLOS reduction strategies. It ensures that patients are discharged as soon as they are medically stable while minimizing the risk of readmission. Discharge planning typically involves comprehensive assessments of the patient’s medical, psychological, and social needs, followed by the coordination of detailed, personalized plans for follow-up care. It has been demonstrated that such discharge planning can help reduce delays in patient discharge and contribute to shorter overall HLOS. A 2022 systematic review concluded that tailored discharge plans are correlated with a small reduction in initial HLOS (mean difference of −0.73 days) and a slight decrease in readmission rates for older patients with medical conditions [96]. Additionally, the study suggests that such discharge planning may slightly enhance patient satisfaction with the healthcare received.

Many hospitals are known to follow a traditional model of discharging patients earlier in the day, particularly before noon [97]. If the patients are being discharged earlier in the day, especially before daily rounds or additional measures, this suggests that patients were stable to be discharged the previous evening or late night. Some studies have argued for making the cutoff according to patients’ age. For example, older patients are more likely not to be discharged late at night when compared to their younger counterparts [98]. These discharge timing patterns reflect a cautious approach aimed at ensuring adequate daytime support and follow-up care upon discharge.

Telehealth follow-ups have emerged as an important component of post-discharge care. By providing virtual check-ins and monitoring, telehealth reduces the need for readmissions and ensures that any complications are promptly addressed. A systematic review found that nurse-led early discharge planning combined with post-discharge telemonitoring reduced readmission rates by 15% and shortened HLOS by an average of 1.2 days [99]. This underscores the importance of follow-up care coordination and adherence in supporting good patient outcomes and lowering hospital burden.

### 3.5. Streamlining Patient Intake and Placement

In addition to these interventions, improving patient placement processes during hospital admissions plays a crucial role in optimizing HLOS. Misallocation of patients to inappropriate hospital units can lead to delays in treatment, prolonged stays, and increased complications. Rathlev et al. (2014) highlighted that ensuring the “right patient, right bed” approach in emergency department admissions significantly reduces placement errors, streamlining care delivery and minimizing unnecessary hospitalization [100]. By refining bed assignment strategies and integrating real-time decision-support tools, hospitals can further enhance patient flow, reduce bottlenecks, and ultimately shorten HLOS. These placement improvements, alongside other targeted interventions, contribute to a more efficient healthcare system while maintaining high-quality patient care.

Interventions aimed at reducing HLOS have shown significant promise in optimizing hospital operations and improving patient outcomes. Enhanced recovery protocols, multidisciplinary teams, outpatient preoperative evaluations, and effective discharge planning are among the most impactful strategies. Incorporating machine learning and other AI tools can significantly reduce HLOS. However, the success of these interventions depends on proper implementation and patient-specific considerations. Future research should focus on tailoring interventions to individual needs and exploring the long-term impact of HLOS reduction on quality of care and healthcare costs.

## 4. Optimizing Hospital Length of Stay: Effective Strategies and Considerations

The literature overwhelmingly supports the significance of HLOS as a key indicator of patient outcomes and hospital efficiency, serving as a proxy for the standard of care and management. Consequently, it is essential for hospitals and healthcare systems to implement effective strategies to mitigate risks associated with prolonged stays. One widely adopted approach is the introduction of Practice Guidelines, which aim to establish standardized protocols for managing patients with similar conditions, thereby minimizing variability in care [101]. While these guidelines help streamline patient management, their rigidity can be a limitation. Since patients respond to treatment differently despite having similar diagnoses, a one-size-fits-all approach may not always be beneficial [102,103]. Additionally, as medical advancements continue to evolve, reliance on outdated guidelines may hinder optimal care if healthcare providers do not adapt their strategies accordingly [104]. Therefore, standardized protocols should be continuously revised based on emerging evidence and tailored to local healthcare contexts rather than strict adherence without flexibility [105].

As previously discussed, ERAS and Fast-Track Rehabilitation programs have emerged as promising strategies to reduce HLOS without increasing adverse events. These programs focus on early mobilization, optimized pain management, and standardized perioperative care. In a systematic review, Greer et al. reported that patients undergoing enhanced recovery programs experienced an average reduction of 2.6 days in HLOS [86]. Similarly, Lee et al. found that implementing an enhanced recovery model in elective gastric cancer surgery reduced HLOS by nearly two days compared to standard recovery pathways, with no increase in post-operative complications [106]. Furthermore, this approach led to a significant reduction in overall hospital costs, reinforcing its potential for both patient benefit and resource optimization. Despite these positive findings, substantial heterogeneity in patient responses suggests that ERAS protocols may need to be adapted to accommodate individual patient needs, particularly for those who may not respond favorably to standardized guidelines.

In addition to ERAS protocols, other strategies such as discharge planning, telehealth, and hospitalist services continue to play a pivotal role in optimizing patient flow and minimizing avoidable days in the hospital. While several strategies have demonstrated efficacy in reducing HLOS, it is equally important to recognize interventions that have not shown significant benefits. For example, a meta-analysis examining the effect of perioperative music therapy on medication requirements and HLOS found promising results in reducing opioid and sedative use, leading to lower medical costs. However, no significant reduction in HLOS was observed [107]. This highlights the need for continued research to distinguish between interventions that improve patient experience and those that directly impact hospital stay duration.

Several strategies have been explored to reduce HLOS, including discharge planning, geriatric assessment, medication management, interdisciplinary or multidisciplinary care, case management, hospitalist services, and telehealth [21,99,108]. While these interventions aim to improve care coordination and facilitate timely discharge, their effectiveness remains inconsistent, particularly in relation to readmissions and mortality across different populations, especially high-risk patients [21]. The variability in outcomes suggests that a standardized approach may not be suitable for all cases, as factors such as patient demographics, comorbidities, and hospital resources play a crucial role in determining success. These findings highlight the need for a more tailored approach, ensuring that interventions are adapted to individual patient needs rather than relying on a universal protocol. Future efforts should focus on refining these strategies to balance reduced HLOS with maintaining high-quality patient care and minimizing adverse outcomes.

## 5. Challenges and Limitations in HLOS Research

HLOS research is subject to significant methodological challenges that hinder the accuracy, comparability, and applicability of findings across healthcare settings. These challenges stem primarily from time-fixed biases, inadequacies in risk adjustment models, inconsistencies in data collection, and heterogeneity in prediction methodologies. Addressing these limitations is crucial for improving HLOS estimation and its use in healthcare decision-making.

### 5.1. Time-Dependent Bias in HLOS Estimation

A fundamental issue in HLOS research is the use of time-fixed statistical methods, which fail to account for the dynamic nature of hospital stays and lead to biased estimates of HLOS attributable to specific conditions. For example, in studies examining healthcare-associated infections (HAIs), conventional time-fixed methods have been shown to overestimate HLOS by an average of 9.4 days—an inflation of 238% compared to time-varying multistate models [109]. Despite this, a majority (75%) of studies continue to use time-fixed approaches, perpetuating this bias [110]. Given that HAIs and other complications occur at varying points during a patient’s stay, applying time-varying methodologies, such as longitudinal and multistate models, is essential to obtain more accurate estimates [111]. Furthermore, other inconsistencies in LOS calculation methods—such as spell-based calculations that underestimate LOS by up to 24%—highlight the need for standardized approaches [112].

### 5.2. Risk Adjustment Model Limitations

Risk adjustment models are integral to HLOS research, yet they frequently demonstrate moderate predictive performance. Studies report that commonly used models have predictive accuracy metrics ranging from 0.30 to 0.60, reflecting limited ability to account for confounding factors [113]. The inclusion of socioeconomic status (SES) and health-related quality of life (HRQOL) data has been shown to enhance model accuracy in an article by Sajobi et al. in 2017; yet, these variables are often missing in HLOS studies [114]. Standardizing the inclusion of these non-clinical variables could improve model reliability and facilitate more accurate comparisons between hospitals.

### 5.3. Challenges in HLOS Prediction Methods

HLOS prediction models have evolved with the advent of machine learning and deep learning techniques; yet, significant methodological limitations persist. While machine learning models offer improved predictive capabilities, their lack of interpretability remains a concern [115]. This is particularly problematic in healthcare, where clinicians must make high-stakes decisions and are hesitant to rely on models that do not provide insight into how predictions are made [115]. Additionally, many prediction models are developed and validated within a single hospital or healthcare system, limiting their generalizability [20]. Standardized performance metrics, rigorous validation protocols, and broader external validation are necessary to enhance the applicability of these models across settings.

### 5.4. Data Standardization and Cross-Setting Compatibility

Data inconsistencies pose a major barrier to accurate HLOS research. Differences in data collection methods, variable definitions, and inclusion criteria across hospitals lead to unreliable comparisons [112]. Moreover, administrative datasets often fail to capture key determinants of HLOS, such as care delays [116]. For example, if a patient needed to be seen by a particular consultant or have a specific specialized test like an MRI or an EEG completed before discharge, this would be important information to know so that consultations and testing can be better streamlined. Likewise, there may be delays in discharge associated with patient placement into various facilities when patients are not returning to their prior living arrangement. Additionally, patients who are undocumented in the United States and do not have health insurance can have significant delays in discharge, sometimes staying in an acute care hospital for over 1 year since they are not able to return to their prior living arrangement but will not be accepted into skilled nursing facilities, acute care rehabilitation facilities, or long-term acute care facilities due to lack of insurance. Establishing standardized HLOS definitions and ensuring the inclusion of comprehensive clinical and non-clinical variables can enhance the robustness of HLOS research.

While the above sections outline key methodological challenges in HLOS research, several areas warrant further exploration. Standardized metrics for future research, particularly those tailored to chronic and psychiatric care settings, remain underdeveloped in the existing literature. Although this section primarily focuses on acute care settings, chronic and psychiatric care may present distinct determinants of HLOS, such as long-term care coordination, psychiatric stabilization, and post-discharge community support. Incorporating specific metrics that account for these contextual differences could provide a more comprehensive understanding of HLOS across diverse patient populations. Additionally, further research is needed to establish standardized guidelines for time-dependent modeling and risk adjustment that accommodate varied healthcare settings, enabling more consistent and actionable findings in HLOS research.

These methodological limitations underscore the need for a more standardized and data-driven approach to HLOS measurement, risk adjustment, and predictive modeling to improve healthcare planning and resource allocation.

### 5.5. Review Limitations

In addition to the methodological challenges discussed above, this review is subject to several limitations that may impact the generalizability and comprehensiveness of its findings. First, the exclusion of grey literature and non-English language studies may have limited the scope of included evidence, potentially omitting relevant studies conducted in non-English-speaking regions or unpublished data. Second, the narrative synthesis approach, while useful for identifying key trends and emerging patterns, is inherently subjective and may introduce bias in the interpretation of findings. Finally, this review does not include a quantitative meta-analysis, limiting the ability to quantitatively assess the strength and consistency of observed associations across studies. Future research should consider incorporating systematic review protocols, meta-analytic techniques, and broader inclusion criteria to address these limitations and enhance the robustness of the evidence base on HLOS determinants and interventions.

## 6. Future Directions and Research Gaps

The transition toward personalized medicine presents valuable opportunities to optimize HLOS by tailoring treatment and recovery plans to individual patients. Research into individualized recovery plans has shown significant potential to enhance both hospital stay duration and patient outcomes. Computational modeling, for instance, has been used to predict unplanned hospital admissions in multimorbid patients, allowing healthcare providers to allocate personalized post-discharge services based on patient profiles. Additionally, precision cohort analytics have improved clinical decision-making by presenting outcomes of past treatment choices for similar patient populations, reinforcing the importance of integrating patient-specific data into care planning [117,118]. These approaches demonstrate how leveraging advanced analytics and predictive modeling can refine HLOS management and improve long-term health outcomes. However, the clinical applicability of such methods remains limited, as real-world evidence on their implementation and sustainability is still emerging.

As the field progresses, future strategies to reduce HLOS are increasingly focusing on integrating real-time monitoring and patient-specific data. Wearable devices and non-invasive digital biomarkers are emerging as tools to monitor health status continuously, particularly in chronic and high-risk conditions where ongoing monitoring aids in early intervention and discharge planning. For instance, motion analysis labs equipped with wearable sensors have been utilized to quantify mobility and quality of life (QoL) in Parkinson’s disease patients, providing objective metrics of disease progression [119]. Similarly, integrating wearable technology in end-of-life care has shown potential in predicting 7-day mortality events in terminal cancer patients, demonstrating how continuous monitoring can support personalized care [117]. Despite the promise of these applications, challenges such as data integration, device standardization, and privacy concerns limit widespread adoption. Addressing these barriers requires more robust real-world evaluations to establish the feasibility and accuracy of wearable technologies in diverse clinical settings [120].

Despite the encouraging preliminary results, the implementation of wearable devices in routine clinical practice remains challenging. For example, the reliability and consistency of digital biomarkers across different devices and patient populations need to be validated. Moreover, integrating these data into existing healthcare workflows without overburdening clinicians requires thoughtful design and thorough testing. As such, further research should focus on large-scale, multi-center trials that can assess the generalizability and long-term impact of wearable monitoring on HLOS reduction.

Beyond the integration of novel technologies, long-term studies are necessary to evaluate the extended effects of HLOS interventions on post-discharge outcomes. Utilizing longitudinal data from electronic health records (EHRs) and continuous monitoring systems may offer insights into the sustained impact of these interventions. For instance, integrating patient-reported outcomes from the PROFILES registry has facilitated the tracking of health trajectories following cancer treatment, highlighting the importance of advanced statistical methods to analyze high-dimensional data [121,122,123]. Further research should prioritize longitudinal data collection and analytics to better understand the long-term implications of HLOS optimization strategies.

Moreover, as AI-driven prediction tools become more sophisticated, there is a pressing need to evaluate their real-world performance in diverse healthcare environments. Emerging studies have begun to assess AI models for HLOS prediction, yet there remains a paucity of data on their comparative effectiveness in clinical practice. For instance, while machine learning algorithms such as Random Forest and Neural Networks have shown potential in controlled settings, their integration into hospital systems requires ongoing validation and adaptation [82]. To maximize clinical utility, future research should focus on comparative studies that benchmark AI model performance against traditional risk stratification methods, providing healthcare stakeholders with evidence-based guidance on model selection and implementation.

Finally, policy frameworks should support the integration of these advanced technologies while prioritizing patient safety and data privacy. Standardizing the use of digital biomarkers and predictive algorithms will require collaboration between technology developers, healthcare providers, and regulatory agencies. Establishing clear guidelines for data sharing, interoperability, and device validation will be crucial for the successful adoption of personalized HLOS interventions.

Grounding future directions in real-world evidence will strengthen the case for personalized and technology-driven approaches to HLOS management. Integrating advanced predictive tools, validating wearable technologies, and addressing policy gaps will be essential for transforming HLOS optimization from a theoretical model to a practical clinical strategy.

## 7. Conclusions

Current evidence underscores the complex relationship between HLOS and patient outcomes, with prolonged stays contributing to increased complications, higher healthcare costs, and greater strain on hospital resources. While patient-specific factors such as comorbidities, demographics, and psychosocial elements play a significant role in HLOS, systemic factors, including hospital management strategies and resource availability, are equally influential.

However, it is essential to recognize that prolonged HLOS is not universally detrimental. In cases involving complex comorbidities or post-operative recovery, extended stays may be necessary to ensure patient safety and optimize long-term outcomes. This nuanced understanding emphasizes the importance of developing HLOS management strategies that balance risks and benefits, particularly for vulnerable populations.

Despite advancements in predictive modeling and machine learning to forecast HLOS, limitations remain regarding model generalizability and the risk of oversimplifying patient complexity. Strategies such as Enhanced Recovery After Surgery (ERAS) protocols and telehealth-based discharge planning have shown promise in reducing HLOS, yet their efficacy varies across populations and healthcare settings. Implementing predictive analytics requires not only methodological rigor but also consideration of ethical implications and patient-centered approaches.

To address the ongoing challenges associated with prolonged HLOS, future research should prioritize the development of targeted interventions that integrate predictive analytics with equitable care frameworks. Additionally, further studies are needed to refine existing models, assess intervention scalability across diverse healthcare settings, and evaluate the impact of HLOS reduction on patient outcomes and healthcare costs. To ensure that such interventions are both effective and equitable, targeted research should explore how predictive models can be tailored to specific patient populations, such as elderly patients with multiple comorbidities or socioeconomically disadvantaged groups. Policymakers and hospital administrators must also consider how disparities in HLOS can be addressed through policies that promote access to post-discharge care and resources for socioeconomically disadvantaged populations. By aligning predictive analytics with value-based care principles, healthcare systems can more effectively balance operational efficiency with patient-centered outcomes, ultimately enhancing care delivery and reducing unnecessary hospital stays.

Ultimately, addressing the complexities of HLOS requires a multifaceted approach that integrates predictive analytics, patient-centered care, and evidence-based policies aimed at reducing disparities and optimizing discharge processes. By advancing these initiatives, healthcare systems can not only improve patient outcomes but also enhance operational efficiency, thereby achieving more sustainable and equitable care delivery.

## Figures and Tables

**Table 1:** Summary of studies on factors influencing hospital length of stay (HLOS).

Study Name	Year	Sample Size	Variables Studied	Impact on HLOS
Major Risk Factors for Mortality in Elderly and Non-Elderly Adult Patients Emergently Admitted for Blunt Chest Wall Trauma [32]	2022	2158	HLOS, comorbidities, mortality	Each additional day: 9% increased mortality (OR = 1.09, *p* = 0.033)
Understanding Variations and Influencing Factors on Length of Stay for T2DM Patients Based on a Multilevel Model [43]	2021	12,888	Gender, insurance type, hospital characteristics, complications	Female: shorter LOS; Elderly: longer LOS (*p* < 0.001)
Factors Contributing to a Longer Length of Stay in Adults Admitted to a Quaternary Spinal Care Center [44]	2023	13,493	Age, admission status, ASIA grade, operative management, adverse events	Advanced age: 1.011/year; Emergency: 1.615 (*p* < 0.05)
Big Data-Driven Determinants of Length of Stay for Patients With Hip Fracture [45]	2020	2238	Gender, age, insurance type, admission route, comorbidities	Comorbidities: significant impact (*p* < 0.05)
Length of Hospital Stay and Associated Factors Among Adult Surgical Patients [46]	2024	452	Referral status, pneumonia, surgery duration, BMI, preoperative anemia	Pneumonia: 3.64; Surgery ≥ 110 min: 2.54 (AOR)
Timing of Regional Analgesia in Elderly Patients with Blunt Chest-Wall Injury Fall [47]	2023	2248	Early vs. late regional analgesia, unplanned intubation, ICU admission, discharge	Early analgesia: 5.5 days vs. 6.5 days (*p* = 0.002)
Orthopedic Pelvic and Extremity Injuries Increase Overall Hospital Length of Stay but Not In-Hospital Complications or Mortality in Trauma ICU Patients [48]	2024	1785	Orthopedic injuries, ICU LOS, hospital LOS, complications, mortality	Orthopedic injury: 1.23 times longer LOS (*p* < 0.001)
Low Falls and Inpatient Complications Increase Risk for Longer Length of Stay in Older Persons Admitted Following Trauma [49]	2025	1250	Delirium, inpatient fall, pneumonia, thromboembolism, blood transfusion, ICU admission	Delirium: IRR 1.41; Inpatient fall: IRR 1.46; Pneumonia: IRR 1.28 (*p* < 0.05)
The Effect of Demographic, Financial and Hospital Factors on the Length of Stay of Preterm Infants [50]	2024	1,359,280	Gestational age (GA), ethnic group, hospital size, geographic region	GA: significant impact; Ethnic group: significant impact (*p* < 0.001)
Risk Factors Associated With Prolonged Hospital Length-of-Stay: 18-Year Retrospective Study of Hospitalizations in a Tertiary Healthcare Center in Mexico [51]	2018	85,904	Age, gender, physician-to-patient ratio, emergency admission, comorbidities	Bone marrow transplant: OR 18.39; Infections: OR 4.65
What Factors Predict Length of Stay in the Intensive Care Unit? Systematic Review and Meta-Analysis [52]	2020	N/A	Mechanical ventilation, hypomagnesemia, delirium, malnutrition	Mechanical ventilation: longer LOS (*p* < 0.05)
Relationships Among Comorbidities, Disease Severity, and Hospitalization Duration in the United States Using the Healthcare Cost and Utilization Project (HCUP) Database [53]	2025	N/A	Age, comorbidities, disease severity	MCCs: >90% elderly; Severe disease: longer LOS
Racial/Ethnic and Socioeconomic Variations in Hospital Length of Stay: A State-Based Analysis [3]	2021	1,432,683	Race/ethnicity, socioeconomic status, median household income by ZIP code	Black patients: 0.25 days longer LOS; Wealthier patients: shorter LOS
Hospital Readmission and Length of Stay Over Time in Patients Undergoing Major Cardiovascular and Orthopedic Surgery: A Tale of 2 States [54]	2016	959,446	CABG, hip and knee replacements, readmission, discharge disposition, mortality	Hip/knee surgery: LOS decreased by 1 day; CABG: unchanged LOS

This table presents key studies examining demographic, clinical, and systemic factors affecting hospital stay duration. It includes study names, sample sizes, primary variables analyzed, and their impact on HLOS. Statistical significance is noted where applicable (e.g., *p*-values or odds ratios). Findings highlight common trends such as increased HLOS with advanced age, comorbidities, and emergency admissions, as well as interventions associated with reduced HLOS.

**Table 2:** Infection rates and increase in HLOS for nosocomial infections.

Infection Type	Infection Rate (%)	Increase in HLOS (Days)
Urinary Tract Infection (UTI)	12.95	3.9
Bloodstream Infection (BSI)	13.00	4.0
Surgical Site Infection (SSI)	9.00	4.7
Pneumonia	15.00	6.6

**Table 3:** Economic burden of prolonged hospitalization associated with nosocomial infections and length of stay (LOS).

Study	Condition	Additional Cost per Patient (USD)
Chen et al. (2005) [69]	Nosocomial Infections	6369
Glied et al. (2016) [57]	Urinary Tract Infections (UTIs)	41,715
	Bloodstream Infections (BSIs)	34,394
	Surgical Site Infections (SSIs)	69,626
	Pneumonia	78,585
Ward et al. (2021) [68]	HLOS Outliers	77,228

Studies are presented by condition, additional cost per patient, and reference. Costs are reported in US dollars (USD).

**Table 4:** Summary of studies examining the association between healthcare conditions, length of stay (LOS), and cost impact.

Study	Population Studied	Length of Stay (LOS)	Cost Impact
Gidey et al. (2023) [70]	Patients with and without HAIs in Ethiopia	Patients with HAIs stayed 8.3 days longer (18.85 vs. 10.59 days)	Average direct medical costs for HAIs were 3033 ETB higher (4826 vs. 1793 ETB)
Taliwal et al. (2025) [71]	Pediatric patients with CKD in the US	LOS for CKD stage 4 and 5 was 56% and 71% longer, respectively	Hospitalization costs were 92% and 147% higher for CKD stage 4 and 5
Monard et al. (2023) [72]	Patients with and without AKI in France	Median LOS for AKI patients was 9 days vs. 0–2 days for non-AKI	Median hospitalization cost was EUR 4719 vs. EUR 735 for non-AKI
Osenenko et al. (2022) [73]	Heart failure (HF) patients in the US	LOS ranged from 3–5 days (median) and 4–7 days (mean)	Cost per hospitalization ranged from USD 7094–USD 9769 (median), USD 10,737–USD 17,830 (mean)
Asegu et al. (2024) [74]	Patients with and without nosocomial infections in Germany	NI patients had a longer LOS by 10 days	Opportunity cost savings of EUR 1000 per preventable NI case
Wakil et al. (2024) [75]	HCC patients in the US	Median LOS increased from 5.79 days (2011) to 6.07 days (2017)	Total charges increased from USD 58,406 (2011) to USD 78,791 (2017)
Liu et al. (2024) [76]	PD patients in Hubei Province, China	Average LOS was 9.9 days	Average cost per patient was USD 1759.9
Dai et al. (2023) [77]	Seniors with mental illness in Dalian, China	Average LOS was 127.51 days	Average hospitalization cost was CNY 33,656.07
Liu et al. (2022) [78]	Patients with and without HAIs in China	Patients with HAIs had 13.89 additional hospitalization days	Total medical expenditure for HAIs was CNY 24,881.37 higher
Chu et al. (2024) [79]	Trauma patients with complications	Complications resulted in increased LOS	Hospitalization costs increased by 1.32-fold with complications
Lv et al. (2023) [80]	Patients with and without HAIs in Western China	Patients with HAIs had significantly higher LOS	Adjusted-discounted costs were significantly higher for HAIs
Gholipour et al. (2023) [81]	COVID-19 patients globally	LOS varied by country and care level	Highest ICU cost per patient was USD 100,789 in Germany

Studies are presented by author, year, population studied, differences in LOS, and financial implications. Costs are reported in original currencies (e.g., ETB = Ethiopian Birr, USD = US dollars, EUR = Euros, CNY = Chinese Yuan).
